# Exposure to the Chinese famine in early life and the risk of anaemia in adulthood

**DOI:** 10.1186/1471-2458-13-904

**Published:** 2013-10-01

**Authors:** Zumin Shi, Cuilin Zhang, Minghao Zhou, Shiqi Zhen, Anne W Taylor

**Affiliations:** 1 Department of Nutrition and Foodborne Disease Prevention, Jiangsu Provincial Center for Disease Control and Prevention, Nanjing, China; 2 Discipline of Medicine, University of Adelaide, 122 Frome Street, Adelaide, SA 5000, Australia; 3 Epidemiology Branch, Division of Epidemiology, Statistics, and Prevention Research, Eunice Kennedy Shriver National Institute of Child Health and Human Development, Bethesda, MD 20852, USA; 4 Jiangsu Provincial Centre for Disease Control and Prevention, 172 Jiangsu Road, Nanjing 210009, China

**Keywords:** Chinese famine, Anemia, Adults, Fetal exposure

## Abstract

**Background:**

Famine exposure during the early stage of life is related to a number of adulthood diseases. The objective of this study was to examine the association of early life exposure to the famine in China (1959–1961) with the risk of anaemia in adulthood.

**Methods:**

We used the data of 2007 adults born between 1954 and 1964 in Jiangsu province from the 2002 Chinese National Nutrition and Health Survey. Anaemia was defined as haemoglobin concentration <12 g/dl in women and <13 g/dl in men.

**Results:**

Prevalence of anaemia in adulthood in nonexposed, fetal-exposed, early-childhood, mid-childhood, and late-childhood exposed to famine groups were 26.0%, 33.8%, 28.1%, 28.2% and 29.7%, respectively. Overall, fetal-exposed to famine was associated with 37% increased risk of anaemia as compared with those non-exposed after adjusting for income, education, place of residence, smoking, alcohol drinking, job, hypertension and BMI; relative risk (95% confidence interval) (RR (95% CI)) was 1.37 (1.09, 1.71). In general, this association appeared to be stronger among men, those who were currently overweight or obese, or those of lower educational levels. Corresponding RR (95% CI) was 1.87 (1.21-2.87), 1.75 (1.20-2.56), and 2.07 (1.37-3.12), respectively.

**Conclusions:**

Fetal exposure to the Chinese famine was associated with an increased risk of anaemia in adulthood.

## Background

The developmental origins hypothesis proposes that under-nutrition in early life is associated with an increased risk of disease in adulthood. Famine studies have provided a number of evidence supporting such a phenomena [1]. The majority of previous famine studies were conducted in developed countries [2,3] and findings were not always consistent with those from developing countries. For example, positive associations of early life famine exposure with metabolic syndrome [4,5] and hypertension [6,7] were found in China and Biafran but not in a Dutch famine study [8].

The Chinese famine of 1959–1961 is the largest one in human history leading to approximately 30 million excess deaths [9,10]. Emerging findings suggested that exposure to Chinese famine in early life was related to elevated risk of diabetes [11], metabolic syndrome [4,5], hypertension [7], short height [12], and overweight [13] in adulthood. Anaemia is a common health problem especially in developing countries [14], with iron deficiency as one of the main causes [14]. Although the prevalence of anaemia has decreased dramatically in China, it is still as high as 15.2% on average [15]. Surprisingly, even in some economically well developed regions, the prevalence of anaemia was above 20% [16] and anaemia often coexists with metabolic syndrome in China [17]. Emerging data suggested that intrauterine exposure to malnutrition increased the risk of anaemia in children [18,19]. However, its implication on anaemia in adulthood has not been investigated and little is known about its long term effect. Investigation of the long-term risk for anemia associated with the Chinese famine will provide a unique opportunity to evaluate fetal origin of adulthood anaemia. Based on data of adults born between 1954 and 1964 in Jiangsu province from the 2002 Chinese National Nutrition and Health Survey, we aimed to investigate whether early life exposure to famine is related to a higher risk of anaemia in adulthood.

## Methods

### Study population

In 2002, China launched a national survey on nutrition and health under the approval of the Chinese Ministry of Health. A multistage cluster sampling method was used to select the participants. The data presented in this article are based on a subsample from Jiangsu province, one of the economically booming areas in China with a population of 73.6 million. The rural sample was selected from six counties in Jiangsu province (Jiangyin, Taicang, Suining, Jurong, Sihong and Haimen). From each of the six counties, three towns were randomly selected. The urban sample was selected from two prefecture capital cities (Nanjing and Xuzhou). From each prefecture city, three streets were randomly selected. The six counties and two prefectures cities represented a geographically and economically diverse population [20]. In each town/street, two villages/neighbourhoods were further randomly selected. In each village/neighbourhood, 90 households were randomly selected. All members of the households were invited to take part in the study. One third of the households were randomly selected to take a detailed survey on food intake. The study was conducted according to the guidelines laid down in the Declaration of Helsinki and all procedures involving human subjects/patients were approved by Jiangsu Provincial Centre for Disease Control and Prevention. Written informed consent was obtained from each participant. In the study presented here, we analyzed only data for adults born between 1952 and 1964. The total analytical population of the present study is composed of 2007 individuals (i.e. 905 men and 1102 women). In the specific analytical population of the present study, famine took place between 1959 and 1961.

### Famine cohort

Five famine cohorts were defined based on date of birth: non-exposed cohort (Oct 1 1962-Sep 30 1964), fetal exposed cohort (Oct 1 1959-Sep 30 1961), early child exposed (Oct 1 1956-Sep 30 1958), mid childhood exposed (Oct 1 1954-Sep 30 1956), and late childhood exposed (Oct 1 1952-Sep 30 1954). Those born between Oct 1 1958 and Sep 30 1959 or between Oct 1 1961 and Sep 30 1962 were excluded in order to minimize misclassification of the famine exposure because the exact start or end date of famine is not clear. Such a classification was used in previous published Chinese famine studies [5,11].

### Measurements

#### ***Haemoglobin***

An overnight fasting blood sample was collected at baseline and follow-up. The blood samples were analyzed for hemoglobin (Hb) by the cyanmethemoglobin method [21] in the local Centers for Disease Control and Prevention according to a standard protocol. Anemia was defined as a Hb level below 13 g/dl for men and 12 g/dl for women [22].

### Anthropometric and other measurements

Anthropometric measurements were obtained using standard protocols and techniques [23,24]. Body weight was measured unshod and in light indoor clothing to the nearest 100 grams with a balance-beam scale, and height to the nearest mm using a stadiometer. Overweight was defined as body mass index (BMI) ≥24 kg/m^2^[25]. Blood pressure was measured twice by mercury sphygmomanometer on the right upper arm of the subject, who was seated for 5 min before the measurement. The mean of these two measurements was used in the analyses. The cuff size was selected on the basis of the upper arm circumference to ensure that the cuff did not overlap [23]. Hypertension was defined as systolic blood pressure above 140 mmHg and/or diastolic blood pressure above 90 mmHg, or use of antihypertensive medications.

### Covariates

Cigarette smoking was assessed by asking the frequency of daily cigarette smoking in the past 30 days. Education was recorded into either 'Low’ (illiteracy, primary school); 'Medium’ (junior middle school); or, 'High’ (high middle school or higher), based on six categories of education levels in the questionnaire. Occupation was recoded into 'Manual’ or 'Non-manual’ based on a question with 12 occupational categories.

### Dietary intake

Nutrients intakes (e.g. iron intake) were assessed using a 3-day weighed food diary which recorded all foods consumed by each individual, on three consecutive days [26].

### Statistical analyses

Chi square test was used to compare differences between categorical variables, and ANOVA was used to compare differences in continuous variables between groups. Poisson regression with robust variance was used to determine the association between famine exposure and anaemia adjusted for education, occupation, smoking, alcohol drinking, hypertension and overweight [27]. We further examined whether the association varied by reported risk factors of anaemia; BMI (i.e. <24 kg/m^2^ vs. ≥24 kg/ m^2^), hypertension (i.e. yes/no), job (manual/non-manual), residence (urban/rural), alcohol drinking (yes/no), smoking (yes/no), income (low/medium/high), and education (low/medium/high) by stratified analyses. Linear association was also used to assess the association between famine exposure and Hb levels. All the analyses were performed using STATA 12 (Stata Corporation, College Station). Statistical significance was considered to be when p < 0.05 (two sided).

## Results

The prevalence of anaemia among adults in late childhood-, mid childhood-, early childhood-, fetal-, and non- exposed cohorts were 29.7, 28.2, 28.1, 33.8, and 26.0%, respectively (Table 1). Compared with the non-exposed cohort, individuals exposed to famine were shorter in height, heavier and more likely to have hypertension than the non-exposed group. Moreover, the prevalence of comorbidity (overweight and anaemia, hypertension and anaemia) was higher among all the famine exposure groups than the non-exposed group. In the fetal exposed group, the prevalence of combined anaemia and overweight and combined anaemia and hypertension was 16.2% and 6.3%, respectively, whereas the corresponding prevalence was 8.7% and 2.7% among the non exposed group, respectively. The prevalence of coexistence of anemia and overweight was higher in all famine exposed groups than non-exposed group (e.g. 16.2% in fetal exposed group, 8.7% in non-exposed group). Furthermore, overweight coexisted in a large percent of study participants who had anaemia, with the highest percent among fetal-exposed to famine group and lowest among non-exposed group (i.e. 48.2% vs. 33.3%). Total iron intake was not significantly different among the different famine exposure groups.

**Table 1:** Characteristics of study population (in 2002) according to famine exposure in early life (N=2007)

	**Exposed cohort**				**Non-exposed cohort**
	**Late childhood**	**Mid childhood**	**Early childhood**	**Fetal-exposed**	
N	468	405	424	272	438
Women (%)	53.0	57.0	52.6	59.9	54.1
Birth date					
(From Oct 1, year)	1952	1954	1956	1959	1962
(To Sep 30, year)	1954	1956	1958	1961	1964
Age in 2002 (years)	48-49	46-47	44-45	41-42	38-39
Anemia (%)	29.7	28.2	28.1	33.8	26.0
Hypertension (%)	29.1	26.4	21.5	21.3	17.8
Hemoglobin (g/dl)	13.3(1.9)	13.3(1.9)	13.5(2.0)	13.2(1.9)	13.5(1.9)
Ferritin (μg/l) ^a^	87.3(83.4)	96.9(92.5)	77.9(65.6)	79.9(83.3)	98.7(90.5)
Height (cm)	160.4(7.7)	160.1(7.8)	160.1(7.8)	160.8(7.3)	162.2(8.0)
Weight (kg)	61.6(9.6)	64.0(47.7)	61.4(10.2)	63.2(10.1)	62.8(11.3)
BMI (kg/m^2^)	23.9(3.1)	24.9(17.1)	23.9(3.4)	24.4(3.3)	23.8(3.3)
Overweight (BMI≥24 kg/m^2^) (%)	45.3	46.3	43.6	49.3	42.7
Comorbidity (%)					
Anemia and overweight	14.3	16.3	13.4	16.2	8.7
Anemia and hypertension	7.9	5.7	6.4	6.3	2.7
Smoker (%)	33.0	28.9	30.8	25.6	27.4
Alcohol drinker (%)	27.7	25.0	31.4	24.7	26.2
Education (%)					
Low	63.7	52.3	42.9	34.2	25.3
Medium	29.1	34.3	33.5	36.4	53.7
High	7.3	13.3	23.6	29.4	21.0
Manual job (%)	62.9	60.2	64.1	60.9	61.1
Living in rural (%)	77.2	72.5	75.2	64.6	65.6
Intake of iron (mg/d) ^a^					
Total iron	25.8(10.9)	27.0(11.2)	27.3(11.0)	26.2(9.9)	27.6(11.7)
Non heme iron	22.9(10.0)	24.0(10.4)	24.6(11.4)	24.0(10.1)	25.1(11.6)
Heme iron	2.9(5.2)	3.0(4.1)	2.7(3.3)	2.3(2.8)	2.5(3.5)

Values are % or mean (SD) unless otherwise specified.

^a^ N=735 (167 in late childhood, 149 in mid childhood, 157 in early childhood, 99 in fetal exposed, 163 in non-exposed cohorts).

Fetal exposure to famine was associated with 37% (95% CI 9%-71%) increased risk of anaemia as compared with non-exposed group after adjusting for income, education, place of residence, smoking, alcohol drinking, job, hypertension and BMI (Table 2). In the fetal exposure cohort, the Hb level was lower than in the non-exposed cohort as indicated by the regression coefficient: regression coefficients were -0.32(-0.71 to 0.06) in men, -0.14(-0.46 to 0.18) and -0.24(-0.49 to 0.00) in men and women combined. In a subsample with dietary data available, further adjustment for dietary intakes of iron did not change the association appreciably (data not shown). In general, this association was appeared to be stronger among men (RR 1.87 95% CI 1.21-2.87) than in women (RR 1.17 95% CI 0.90-1.53) although the test for interaction was not statistically significant.

**Table 2:** Association of famine exposure during early life with risk of adulthood anemia in Chinese (n=2007)

	**Exposed cohorts**				**Nonexposed cohort**
	**Late childhood**	**Mid childhood**	**Early childhood**	**Fetal-exposed**	
*Anemia as outcome*					
Both genders					
Prevalence	29.7	28.2	28.1	33.8	26.0
RR (95% CI) : model 1	1.15(0.93-1.41)	1.07(0.86-1.33)	1.09(0.88-1.35)	**1.26(1.01-1.59)**	1.00
RR (95% CI) : model 2	1.05(0.84-1.30)	1.02(0.81-1.28)	1.04(0.84-1.29)	**1.36(1.08-1.70)**	1.00
RR (95% CI) : model 3	1.05(0.84-1.30)	1.03(0.82-1.29)	1.04(0.84-1.30)	**1.37(1.09-1.71)**	1.00
Men					
Prevalence	23.6	21.8	17.9	28.4	16.4
RR (95% CI) : model 1	1.44(0.97-2.13)	1.33(0.87-2.02)	1.09(0.71-1.68)	**1.73(1.13-2.67)**	1.00
RR (95% CI) : model 2	1.13(0.74-1.74)	1.16(0.74-1.83)	1.02(0.65-1.59)	**1.80(1.17-2.77)**	1.00
RR (95% CI) : model 3	1.17(0.76-1.79)	1.19(0.76-1.86)	1.02(0.66-1.60)	**1.87(1.21-2.87)**	1.00
Women					
Prevalence	35.1	32.9	37.2	37.4	34.2
RR (95% CI) : model 1	1.03(0.80-1.31)	0.96(0.75-1.24)	1.09(0.85-1.39)	1.09(0.84-1.43)	1.00
RR (95% CI) : model 2	0.96(0.75-1.24)	0.94(0.72-1.22)	1.04(0.81-1.34)	1.16(0.88-1.51)	1.00
RR (95% CI) : model 3	0.98(0.76-1.26)	0.95(0.73-1.24)	1.06(0.82-1.36)	1.17(0.90-1.53)	1.00

Model 1: unadjusted model.

Model 2: RR adjusted for income, education, residence (urban/rural), smoking, alcohol drinking, job (manual vs non manual), hypertension. In gender combined model, gender was also adjusted in the model.

Model 3: additional adjustment for BMI.

In stratified analyses, in general, the elevated risk of adulthood anaemia associated with fetal exposure to famine persisted across different stratum of other risk factors of anaemia, such as overweight status (yes/no), hypertension (yes/no), smoking (yes/no), income (low/medium/high), and education (low/medium/high) although it became statistically insignificant in some strata due to the reduced sample size. The association seemed strongest among those with low education or overweight/obese.

Adjusted RR (95% CI) of fetal-exposed to famine in association with anaemia in adulthood was 1.75 (1.20-2.56) for overweight or obese individuals and 1.17 (0.88-1.57) for others; 2.07 (1.37-3.12) for those of low education vs 1.34 (0.93-1.93) for others.

When we limit our analyses to the rural sample, the RRs for anemia were 1.25(0.96-1.61), 1.05(0.80-1.38), 1.18(0.91-1.52), 1.49(1.13-1.96), and 1.00 for late childhood, mid childhood, early childhood, fetal-exposed and non-exposed cohorts, respectively. There was significant interaction between residence (urban/rural) and famine exposure (p=0.023). Among urban residents, there was no significant association between famine exposure and anemia (data not shown). In fully adjusted regression model using the rural sample, the regression coefficients for Hb were -0.22 (-0.48 to 0.03), -0.03(-0.28 to 0.23), -0.07(-0.32 to 0.18), -0.34(-0.63 to -0.04) and 0.00 for late childhood, mid childhood, early childhood, fetal-exposed and non-exposed cohorts, respectively.

## Discussion

We found that fetal exposure to the Chinese famine was associated with an increased risk of anaemia especially among men, those currently overweight or obese or with lower educational levels. Moreover, coexistence of anaemia and overweight/hypertension was more common among those exposed to famine than those non-exposed.

We are unaware of previous studies assessing early life exposure to famine and anaemia in adulthood. Several factors related to body iron levels and store may be implicated in the observed association between famine exposure in early life and adulthood anaemia, for instance dietary iron intakes. However, in a subsample with data on dietary iron intakes available, we observed that there is no significant difference in iron intake across different famine groups. Therefore, the difference in the prevalence of anemia between famine exposure groups and non-exposed cannot be explained by differences in iron intake. Similar phenomenon was found in a study in Mexico [28], which found iron deficiency in obese women and children were predicted by obesity-related inflammation rather than by differences in dietary iron intake. During famine, it would be expected that the level of stress will be high. In an animal study it was found that maternal stress during pregnancy predisposes for iron deficiency in infant monkeys [29]. In a recent study of 140 pregnant women in Israel, it was found that prenatal maternal stress (under rocket attack during a military operation) predicts lower cord ferritin concentration [30]. However, whether this effect can last until adulthood is not known.

The association between fetal-exposed to famine and anaemia appeared to be more evident among men than women. The underlying mechanisms are unclear. On one hand, famine exposure was related to a lower sex ratio leading to more female babies [31], suggesting women may be more adaptable to famine. On the other hand, menstruation, specific to women, is an important factor regulating haemoglobin levels. As we do not have information on menstruation and menopasue, we are not able to control the effect of blood loss due to menstruation. It has been reported that there was a positive association between testosterone and haemoglobin levels among men [32]. Whether a low testosterone level mediates the association between famine exposure and anemia among men warrants further investigation. In addition to differential effect by gender, in the present study, the association of fetal-exposed to famine with adulthood anaemia appeared strongest among the low socioeconomic status (SES). It is plausible that those in the low SES group suffered most from famine due to poor access to other resources. Those exposed prenatally will also have been exposed in early life since the famine lasted for 3 years rather than 9 months. The effects of exposure in pregnancy only are thus difficult to assess.

During famine, there was a significant drop in fertility. In the study area, the total fertility loss during famine was 128.3% [33]. Those who chose to give birth during famine would be those with the capacity to cope with the famine. In the study area, the excess crude death rate was 11.375 per 1000 during the famine period [33]. Because of the long duration of the famine, children born during the famine would suffer from both prenatal and postnatal under-nutrition and lead to both anemia and high mortality. Children with worse health condition would be more likely to die from famine than those with better health. Thus, both selective fertility and selective mortality would underestimate the true effect of famine in the population.

Comorbidity of anaemia and being overweight was common in famine exposed groups in this population. Among those who are anemic, the prevalence of overweight differs between fetal exposure and non-exposed cohorts (48.2% vs 33.3%). It is possible that anaemia secondary to chronic diseases is a problem in this population. Due to the lack of biomarkers of chronic diseases such as inflammation, we are not able to further explore this hypothesis. Findings from our previous studies on the association between diet and anaemia indicated that inflammation may play a role in the aetiology of anaemia in the population [26]. Future study should assess whether famine exposure affect biomarkers of inflammation such as C-reactive protein.

The study has several strengths. Firstly, it is based on a large sample composed of both urban and rural populations following standard manual of procedures. Anthropometric measurements were performed by health workers during a household visit according to a standard protocol. Other major characteristics of study participants were collected based on in person interview according to a standard protocol, which may reduce the measurement errors. Secondly, in a subsample, we have detailed information on dietary intakes. Several limitations of the study merit discussion. First we do not have information on iron metabolism biomarkers, and some other factors that may affect body iron store and levels such as menstruation status in women that are related to the development of anaemia. Secondly, regional difference in the severity of famine is highly likely in the study population, as the neighbouring province, Anhui, had an excess death rate of 474.9% during the famine period [10]. We are not able to assess the effect of the severity of famine in association with anemia due to small number of participants in the fetal- exposed group. In sensitivity analysis, when we subdivided the sample into subgroups, the power of detecting a significant association in some subgroups may be limited given the relatively small number in certain subgroups. The findings from stratified analyses are suggestive not conclusive. Selection bias is possible due to selective mortality or reduced fertility across famine exposure groups especially in the fetal exposed group.

## Conclusion

In conclusion, we observed that early life exposure to the Chinese famine was associated with an increased risk of anaemia particularly among men, those currently overweight or obese, or with low education levels. These findings suggested that both the early life under nutrition and later life over nutrition are related to health condition in adulthood. The underlying molecular mechanisms warrant future investigation.

## Abbreviations

BMI: Body mass index; Hb: Haemoglobin; SES: Socio-economic status.

## Competing interests

The authors declare that they have no competing interests.

## Authors’ contributions

ZS conceived the study. ZS and CZ completed all the statistical analyses, and drafted the manuscript. MZ, SZ and AWT contributed to the discussion. All authors have read and approved the final manuscript.

## Pre-publication history

The pre-publication history for this paper can be accessed here:

http://www.biomedcentral.com/1471-2458/13/904/prepub
